# On estimation of *P*(*Y* < *X*) for inverse Pareto distribution based on progressively first failure censored data

**DOI:** 10.1371/journal.pone.0287473

**Published:** 2023-11-30

**Authors:** Randa Alharbi, Renu Garg, Indrajeet Kumar, Anita Kumari, Ramy Aldallal

**Affiliations:** 1 Department of Statistics, Faculty of Science, University of Tabuk, Tabuk, Saudia Arabia; 2 Department of Statistics, Kirori Mal College, University of Delhi, Delhi, India; 3 Department of Mathematics, Kalasalingam Academy of Research and Education, Krishnankoil, Tamilnadu, India; 4 Department of Statistics, Central University of Haryana, Mahendergarh, India; 5 Department of Accounting, College of Business Administration in Hawtat Bani Tamim, Prince Sattam Bin Abdulaziz University, Jeddah, Saudi Arabia; University of Eastern Finland, FINLAND

## Abstract

The stress-strength reliability (SSR) model *ϕ* = *P*(*Y* < *X*) is used in numerous disciplines like reliability engineering, quality control, medical studies, and many more to assess the strength and stresses of the systems. Here, we assume *X* and *Y* both are independent random variables of progressively first failure censored (PFFC) data following inverse Pareto distribution (IPD) as stress and strength, respectively. This article deals with the estimation of SSR from both classical and Bayesian paradigms. In the case of a classical point of view, the SSR is computed using two estimation methods: maximum product spacing (MPS) and maximum likelihood (ML) estimators. Also, derived interval estimates of SSR based on ML estimate. The Bayes estimate of SSR is computed using the Markov chain Monte Carlo (MCMC) approximation procedure with a squared error loss function (SELF) based on gamma informative priors for the Bayesian paradigm. To demonstrate the relevance of the different estimates and the censoring schemes, an extensive simulation study and two pairs of real-data applications are discussed.

## 1 Introduction

The stress-strength reliability (SSR) analysis is a statistical measurement of the interaction between the component’s strength and the stresses applied to it on a system. SSR analysis is a popular statistical tool used in reliability engineering that is useful in many disciplines such as medical studies, engine aircraft testing, physical strength testing of buildings or bridges, and so on. Assume that *X* and *Y* are random variables measuring the strength and stress of a system, respectively. Then the system’s SSR is described as *ϕ* = *P*(*Y* < *X*), and the system will fail if *X* ≤ *Y*. This concept was first suggested by [1], who demonstrated how the Mann-Whitney statistic *U* could be used to estimate SSR *ϕ* with given observations *Y*_1_, *Y*_1_, …, *Y*_*n*_; *X*_1_, *X*_2_, …, *X*_*m*_ from continuous populations. Specifically, he proposed the SSR $\phi =\frac{U}{mn}$. Since then, this concept has been widely adopted in many real-world applications. For example, [2] used the SSR concept in military applications, [3] discussed various SSR models and their applications. Several authors also investigated SSR models for different lifetime models using both complete and censored samples. Some recent work based on complete samples are discussed in the following studies: [4–6]. A number of authors have contributed works to the censored life test scenarios in literature and the work based on different censored samples are given by [7–13], etc.

We require data on variables *X* and *Y* to estimate SSR. Observed data are usually gathered through a life test. In life testing experiments, researchers plan some failures but due to test anomalies, equipment failures, and operating errors, they do not get failures as expected. Also, they have to finish their experiment before all the experimental units are exhausted due to limited budget or due to shortage of time. In such cases, they get censored data rather than the complete sample data. In real life, there are situations where researchers have to remove live units during the experiment, then the progressive first failure censoring scheme (PFFCS) is the best choice. The PFFCS has become the most popular censoring scheme in the last decade as it allows the intermittent removal of the live units from the experiment. When the tested items in a large batch are less costly or the inspection cost is high, the PFFCS is popularly used. The PFFCS was proposed by [14]. They showed PFFCS exhibits some special behaviors to the other censoring schemes. Due to these flexible behaviors, a lot of coverage with several applications has appeared in the literature in the last decade, for example, [15–17]. This is how the PFFCS is explained: Place *n* independent groups with *k* test items in a life test, and the test will be ended whenever a prefixed number of failures (*m*) has been met. Failures are gathered in the following way:

- As soon as the first failure (*X*_1:*m*:*n*:*k*_) happens, remove *G*_1_ live groups at random, and the group that contains *X*_1:*m*:*n*:*k*_ from the test.
- As soon as the first failure (*X*_2:*m*:*n*:*k*_) happens, remove *G*_2_ live groups at random, and the group that contains *X*_2:*m*:*n*:*k*_ from the test and so on.
- Finally, as soon as *m*^*th*^ failure (*x*_*m*:*m*:*n*:*k*_) occurs, remaining *G*_*m*_ live groups along with group contains *x*_*m*:*m*:*n*:*k*_ are removed from the test.

In this way, *X*_1:*m*:*n*:*k*_ < *X*_2:*m*:*n*:*k*_ < … < *X*_*m*:*m*:*n*:*k*_, is observed as ordered progressively first failure-censored (PFFC) sample with prefixed censoring plans $\underset{˜}{G}=\left(G_{1},G_{2},\ldots ,G_{m}\right)$, where *n* = *m* + *G*_1_ + *G*_2_ + … + *G*_*m*_.

If the failure times under the test have a continuous pdf *f*(*x*|Θ) and cdf *F*(*x*|Θ), the joint pdf for *X*_1:*m*:*n*:*k*_ < *X*_2:*m*:*n*:*k*_ < … < *X*_*m*:*m*:*n*:*k*_ is given as follows:

$$\begin{array}{c} f_{\underset{∼}{X}}\left(x_{1},x_{2},..,x_{m}|\Theta \right)=Ck^{m}\prod_{j=1}^{m}\;f\left(x_{j:m:n:k}|\Theta \right){\left(1-F\left(x_{j:m:n:k}|\Theta \right)\right)}^{k(G_{j}+1)-1} \end{array}$$ (1)

$$0<x_{1}<x_{2}<\ldots <x_{m}<\infty ,$$

where Θ is the parameters space and *C* = *n*(*n* − *G*_1_ − 1)(*n* − *G*_1_ − *G*_2_ − 2)…(*n* − *G*_1_ − *G*_2_ − … − *G*_*m*−1_ − *m* + 1). The following censoring schemes are special cases of PFFCS:

(i) The PFFC sample reduces to first-failure censored data when $\underset{˜}{G}=0,0,\ldots ,0$.
(ii) The PFFC sample reduces to progressively type-II censored data when *k* = 1.
(iii) The PFFC sample becomes Type-II censored sample when *k* = 1 and $\underset{˜}{G}=0,0,\ldots ,n-m$.
(iv) The PFFC sample becomes the complete sample case when *k* = 1 and $\underset{˜}{G}=0,0,\ldots ,0$.

## 2 Model description and SSR

The inverse Pareto distribution (IPD) is a one-parameter lifetime distribution that has two different shapes, an upside-down bathtub and decreasing, of the failure rate function. In real-life applications, there are many situations where both failure rate functions are very useful. The IPD is adaptable to the various failure rate function forms that are frequently observed in medical studies such as cancer data, heart transplant data, etc. To analyze such data IPD may be appropriate, see [18]. In reliability engineering, the application of the IPD lifetime model has been discussed by [4] with the help of the failure time of the air conditioning system of two airplanes. Also, a number of research papers have described the popularity of the IPD lifetime model. For example, [19] discussed several estimations of parameter and reliability characteristics with the application of head-neck cancer data and also compared the performance of the IPD lifetime model with some other existing lifetime models, and [12] discussed SSR based on progressively censored data for IPD lifetime model. [20] studied reliability estimation using progressively first-failure censored data. Such behavior of the IPD lifetime model and its diverse applications motivate us to contribute some more ideas in reliability engineering.

Let *X* be a random variable with IPD having a probability density function (pdf) *f*_*X*_(*x*|*α*), and a cumulative distribution function (cdf) *F*_*X*_(*x*|*α*), respectively

$$\begin{array}{c} f_{X}\left(x|\alpha \right)=\frac{\alpha x^{\alpha -1}}{{\left(1+x\right)}^{\alpha +1}};\;x>0,\alpha >0, \end{array}$$ (2)

$$\begin{array}{c} F_{X}\left(x|\alpha \right)={\left(\frac{x}{1+x}\right)}^{\alpha };\;x>0,\alpha >0. \end{array}$$ (3)

Here, *α* is a scale parameter. The goal of this paper is to develop maximum product spacing (MPS) method for the SSR of IPD based on PFFC data. In the literature, the MPS method has not yet been investigated for PFFC data. Also, we considered maximum likelihood (ML) and Bayesian estimation methods to construct the SSR. Let *X* and *Y* be independent random variables following IPD(*α*_1_) and IPD(*α*_2_), respectively, then the SSR is defined as

$$\phi =P(Y<X)=\int_{0}^{\infty }F_{X}\left(x\right)f_{X}\left(x\right)dx=\frac{\alpha_{1}}{\alpha_{1}+\alpha_{2}}.$$ (4)

The rest of the article is laid out as follows: The MPS and ML methods for estimating SSR are addressed in Section 3. The interval estimate of SSR based on ML estimate is also discussed. The Bayes estimator and their corresponding interval estimate of SSR are discussed in Section 4. Section 5, To assess the efficiency of the SSR estimators, a comprehensive simulation study is carried out. A pair of real data sets are analyzed in Section 6 to illustrate the suggested technique. Finally, a concluding remark appears in Section 7.

## 3 Classical estimation

The SSR is estimated in this section using two estimation procedures: maximum product spacing (MPS) and maximum likelihood (ML) estimation methods.

### 3.1 Maximum product spacing method

Here, we discuss the maximum product spacing (MPS) method to estimate the SSR *ϕ*. Initially, this method was first introduced by [21], which was then employed by [22], they showed it is an alternative to the ML estimation method. In addition, the MPS method also follows consistency, asymptotic, and invariance properties similar to those of the ML estimation method. However, in the ML estimation method, parameter values are chosen to maximize the likelihood function, but in the MPS method, parameter values are chosen to maximize the product of the gaps between the values of the distribution function at adjacent ordered points. [23] recently recommended the use of the MPS technique for progressively censored data, which selects the parameter values that make the observed data as uniform as possible.

However, in this study, we proposed to generalize [23] recommended MPS method for progressively censored data to PFFC data. The product of spacing to be maximized based on PFFC data can be defined as follows: Let $x_{i:m_{1}:n_{1}:k_{1}};i=1,2,\ldots ,m_{1}$ be a PFFC sample obtained from *n*_1_ testing groups each having *k*_1_ units with pre-fixed censoring scheme $\underset{˜}{G}=\left(G_{1},G_{2},\ldots ,G_{m_{1}}\right)$ from IPD(*α*_1_). Similarly, let $Y_{j:m_{2}:n_{2}};j=1,2,\ldots ,m_{2}$ be a PFFC sample obtained from *n*_2_ testing groups each having *k*_2_ units with pre-fixed censoring scheme $\underset{˜}{W}=\left(W_{1},W_{2},\ldots ,W_{m_{2}}\right)$ from IPD(*α*_2_). Then, the product spacing’s are defined as

$$\begin{array}{cc} Q(\alpha_{1},\alpha_{2})= & \prod_{i=1}^{m_{1}+1}[F_{X}\left(x_{i};\alpha_{1}\right)-F_{X}\left(x_{i-1};\alpha_{1}\right)]{\left[1-F_{X}\left(x_{i};\alpha_{1}\right)\right]}^{k_{1}\left(G_{i}+1\right)-1}\\ \times & \prod_{j=1}^{m_{2}+1}[F_{Y}\left(y_{j};\alpha_{2}\right)-F_{Y}\left(y_{j-1};\alpha_{2}\right)]{\left[1-F_{Y}\left(y_{j};\alpha_{2}\right)\right]}^{k_{2}\left(W_{j}+1\right)-1}, \end{array}$$ (5)

where,

$$F_{X}\left(x_{0:m_{1}:n_{1}:k_{1}}\right)≃0,\;\;F_{X}\left(x_{m_{1}+1:m_{1}:n_{1}:k_{1}}\right)≃1$$

$$F_{Y}\left(y_{0:m_{2}:n_{2}:k_{2}}\right)≃0,\;\;F_{Y}\left(y_{m_{2}+1:m_{2}:n_{2}:k_{2}}\right)≃1.$$

Thus, using Eq (3) in Eq (5), the product spacing’s are given by

$$\begin{array}{cc} Q\left(\alpha_{1},\alpha_{2}\right) & =\prod_{i=1}^{m_{1}+1}[{\left(\frac{x_{i}}{1+x_{i}}\right)}^{\alpha_{1}}-{\left(\frac{x_{i-1}}{1+x_{i-1}}\right)}^{\alpha_{1}}]\prod_{i=1}^{m_{1}+1}{\left[1-{\left(\frac{x_{i}}{1+x_{i}}\right)}^{\alpha_{1}}\right]}^{k_{1}\left(G_{i}+1\right)-1}\\ & \;\;\;\times \prod_{j=1}^{m_{2}+1}\left[{\left(\frac{y_{j}}{1+y_{j}}\right)}^{\alpha_{2}}-{\left(\frac{y_{j-1}}{1+y_{j-1}}\right)}^{\alpha_{2}}\right]\prod_{j=1}^{m_{2}+1}{\left[1-{\left(\frac{y_{j}}{1+y_{j}}\right)}^{\alpha_{2}}\right]}^{k_{2}\left(W_{j}+1\right)-1} \end{array}$$ (6)

Taking natural logarithm, *H* = ln *Q*(*α*_1_, *α*_2_), of Eq (6) we get

$$\begin{array}{cc} H & =\sum_{i=1}^{m_{1}+1}\;\text{ln}\;\left[{\left(\frac{x_{i}}{1+x_{i}}\right)}^{\alpha_{1}}-{\left(\frac{x_{i-1}}{1+x_{i-1}}\right)}^{\alpha_{1}}\right]+\sum_{i=1}^{m_{1}}\left[k_{1}\left(G_{i}+1\right)-1\right]\;\text{ln}\;\left[1-{\left(\frac{x_{i}}{1+x_{i}}\right)}^{\alpha_{1}}\right]\\ & +\sum_{j=1}^{m_{2}+1}\;\text{ln}\;\left[{\left(\frac{y_{j}}{1+y_{j}}\right)}^{\alpha_{2}}-{\left(\frac{y_{j-1}}{1+y_{j-1}}\right)}^{\alpha_{2}}\right]+\sum_{j=1}^{m_{2}}\left[k_{2}\left(W_{j}+1\right)-1\right]\;\text{ln}\;\left[1-{\left(\frac{y_{j}}{1+y_{j}}\right)}^{\alpha_{2}}\right]. \end{array}$$ (7)

The following normal equations are obtained by differentiating Eq (7) w.r.t *α*_1_ and *α*_2_, respectively:

$$\begin{array}{cc} H_{\alpha_{1}}^{\prime } & =\sum_{i=1}^{m_{1}+1}\frac{1}{A_{1}}\{{\left(\frac{x_{i}}{1+x_{i}}\right)}^{\alpha_{1}}\;\text{ln}\;\left(\frac{x_{i}}{1+x_{i}}\right)-{\left(\frac{x_{i-1}}{1+x_{i-1}}\right)}^{\alpha_{1}}\;\text{ln}\;\left(\frac{x_{i-1}}{1+x_{i-1}}\right)\}\\ & +\sum_{i=1}^{m_{1}}\;\left[k_{1}\left(G_{i}+1\right)-1\right]\frac{1}{B_{1}}{\left(\frac{x_{i}}{1+x_{i}}\right)}^{\alpha_{1}}\;\text{ln}\;\left(\frac{x_{i}}{1+x_{i}}\right)=0 \end{array}$$ (8)

and

$$\begin{array}{cc} H_{\alpha_{2}}^{\prime } & =\sum_{j=1}^{m_{2}+1}\frac{1}{A_{2}}\{{\left(\frac{y_{j}}{1+y_{j}}\right)}^{\alpha_{2}}\;\text{ln}\;\left(\frac{y_{j}}{1+y_{j}}\right)-{\left(\frac{y_{j-1}}{1+y_{j-1}}\right)}^{\alpha_{2}}\;\text{ln}\;\left(\frac{y_{j-1}}{1+y_{j-1}}\right)\}\\ & +\sum_{j=1}^{m_{2}}\;\left[k_{2}\left(W_{j}+1\right)-1\right]\frac{1}{B_{2}}{\left(\frac{y_{j}}{1+y_{j}}\right)}^{\alpha_{2}}\;\text{ln}\;\left(\frac{y_{j}}{1+y_{j}}\right)=0, \end{array}$$ (9)

where,

$$A_{1}=\left[{\left(\frac{x_{i}}{1+x_{i}}\right)}^{\alpha_{1}}-{\left(\frac{x_{i-1}}{1+x_{i-1}}\right)}^{\alpha_{1}}\right],\;\;B_{1}=\left[1-{\left(\frac{x_{i}}{1+x_{i}}\right)}^{\alpha_{1}}\right]$$

$$A_{2}=\left[{\left(\frac{y_{j}}{1+y_{j}}\right)}^{\alpha_{2}}-{\left(\frac{y_{j-1}}{1+y_{j-1}}\right)}^{\alpha_{2}}\right],\;\;B_{2}=\left[1-{\left(\frac{y_{j}}{1+y_{j}}\right)}^{\alpha_{2}}\right].$$

The MPS estimates say (${\tilde{\alpha }}_{1},{\tilde{\alpha }}_{2}$) of (*α*_1_, *α*_2_) are the solutions of (8) and (9), respectively. For Eqs (8) and (9), there are no closed-form solutions available. An appropriate iterative approach can be utilized to get numerical solutions to these nonlinear equations. After obtaining MPS estimates of *α*_1_ and *α*_2_, the MPS estimate of SSR, say $\tilde{\phi }$, is calculated using the invariance property of MPS estimators and is provided by

$$\begin{array}{c} \tilde{\phi }=\frac{{\tilde{\alpha }}_{1}}{{\tilde{\alpha }}_{1}+{\tilde{\alpha }}_{2}}. \end{array}$$ (10)

### 3.2 Maximum likelihood estimation method

In this subsection, the estimate of SSR *ϕ* is obtained using the ML estimation method. Let $x_{i:m_{1}:n_{1}k_{1}};i=1,2,\ldots ,m_{1}$, be independent PFFC censored sample from IPD(*α*_1_) with presumed censoring plan $\underset{˜}{G}=\left(G_{1},G_{2},\ldots ,G_{m_{1}}\right)$ and similarly let $Y_{j:m_{2}:n_{2}:k_{2}};j=1,2,\ldots ,m_{2}$ be independent PFFC sample from IPD(*α*_2_) with presumed censoring scheme $\underset{˜}{W}=\left(W_{1},W_{2},\ldots ,W_{m_{2}}\right)$, then using Eqs (2), (3) and (1), the likelihood function is given by

$$\begin{array}{cc} L\left(\alpha_{1},\alpha_{2};\underset{˜}{x},\underset{˜}{y}\right) & =C_{1}C_{2}k_{1}^{m_{1}}k_{2}^{m_{2}}\prod_{i=1}^{m_{1}}\;f_{X}\left(x_{i}\right){\left[1-F_{X}\left(x_{i}\right)\right]}^{k_{1}\left(G_{i}+1\right)-1}\\ & \;\;\times \prod_{j=1}^{m_{2}}\;f_{Y}\left(y_{j}\right){\left[1-F_{Y}\left(y_{j}\right)\right]}^{k_{2}\left(W_{i}+1\right)-1}\\ & =C_{1}C_{2}k_{1}^{m_{1}}k_{2}^{m_{2}}\alpha_{1}^{m_{1}}\alpha_{2}^{m_{2}}\prod_{i=1}^{m}\frac{x_{i}^{\alpha_{1}-1}}{{\left(1+x_{i}\right)}^{\alpha_{1}+1}}{\left[1-{\left(\frac{x_{i}}{1+x_{i}}\right)}^{\alpha_{1}}\right]}^{k_{1}\left(G_{i}+1\right)-1}\\ & \;\times \prod_{j=1}^{m_{2}}\frac{y_{j}^{\alpha_{2}-1}}{{\left(1+y_{j}\right)}^{\alpha_{2}+1}}{\left[1-{\left(\frac{y_{j}}{1+y_{j}}\right)}^{\alpha_{2}}\right]}^{k_{2}\left(W_{j}+1\right)-1} \end{array}$$ (11)

where, $C_{1}=n_{1}(n_{1}-G_{1}-1)(n_{1}-G_{1}-G_{2}-2)\ldots (n_{1}-G_{1}-G_{2}-\cdots -G_{m_{1}-1}-m_{1}+1)$ and $C_{2}=n_{2}(n_{2}-W_{1}-1)(n_{2}-W_{1}-W_{2}-2)\ldots (n_{2}-W_{1}-W_{2}-\cdots -W_{m_{2}-1}-m_{2}+1)$. The corresponding log-likelihood function is obtained as

$$\begin{array}{cc} l\left(\alpha_{1},\alpha_{2}\right) & =D+m_{1}\;\text{ln}\;\alpha_{1}+\alpha_{1}\sum_{i=1}^{m_{1}}\;\text{ln}\;\left(\frac{x_{i}}{1+x_{i}}\right)+\sum_{i=1}^{m_{1}}\;\left[k_{1}\left(G_{i}+1\right)-1\right]\;\text{ln}\;\left[1-{\left(\frac{x_{i}}{1+x_{i}}\right)}^{\alpha_{1}}\right]\\ & \;\;+m_{2}\;\text{ln}\;\alpha_{2}+\alpha_{2}\;\sum_{j=1}^{m_{2}}\;\text{ln}\;\left(\frac{y_{i}}{1+y_{i}}\right)+\sum_{j=1}^{m_{2}}\;\left[k_{2}\left(W_{j}+1\right)-1\right]\;\text{ln}\;\left[1-{\left(\frac{y_{j}}{1+y_{j}}\right)}^{\alpha_{2}}\right], \end{array}$$ (12)

where, $D=\text{ln}\left(C_{1}C_{2}\right)+m_{1}\;\text{ln}\;k_{1}+m_{2}\;\text{ln}\;k_{2}-\sum_{i=1}^{m_{1}}\;\text{ln}\;\left[x_{i}\left(1+x_{i}\right)\right]-\sum_{j=1}^{m_{2}}\;\text{ln}\;\left[y_{j}\left(1+y_{j}\right)\right]$. The normal equations are obtain by differentiating (12) w.r.t *α*_1_ and *α*_2_, respectively

$$\begin{array}{c} \frac{\partial l\left(\alpha_{1},\alpha_{2}\right)}{\partial \alpha_{1}}=\frac{m_{1}}{\alpha_{1}}+\sum_{i=1}^{m_{1}}\;\text{ln}\;\left(\frac{x_{i}}{1+x_{i}}\right)-\sum_{i=1}^{m_{1}}\;\left[k_{1}\left(G_{i}+1\right)-1\right]\frac{{\left(\frac{x_{i}}{1+x_{i}}\right)}^{\alpha_{1}}\;\text{ln}\;\left(\frac{x_{i}}{1+x_{i}}\right)}{\left[1-{\left(\frac{x_{i}}{1+x_{i}}\right)}^{\alpha_{1}}\right]}=0. \end{array}$$ (13)

and,

$$\begin{array}{c} \frac{\partial l\left(\alpha_{1},\alpha_{2}\right)}{\partial \alpha_{2}}=\frac{m_{2}}{\alpha_{2}}+\sum_{j=1}^{m_{2}}\;\text{ln}\;\left(\frac{y_{j}}{1+y_{j}}\right)-\sum_{j=1}^{m_{2}}\;\left[k_{2}\left(W_{j}+1\right)-1\right]\frac{{\left(\frac{y_{j}}{1+y_{j}}\right)}^{\alpha_{2}}\;\text{ln}\;\left(\frac{y_{j}}{1+y_{j}}\right)}{\left[1-{\left(\frac{y_{j}}{1+y_{j}}\right)}^{\alpha_{2}}\right]}=0. \end{array}$$ (14)

The solution of normal Eqs (13) and (14) are the ML estimates ($\hat{\alpha_{1}}$, $\hat{\alpha_{2}}$) of (*α*_1_, *α*_2_). Here, the closed form solutions for (13) and (14) are unavailable. An appropriate iterative approach can be applied to get numerical solutions to these non-linear equations. The ML estimate of SSR, say $\hat{\phi }$ is computed using the invariance property of ML estimators and is given by

$$\begin{array}{c} \hat{\phi }=\frac{\hat{\alpha_{1}}}{\hat{\alpha_{1}}+\hat{\alpha_{2}}}. \end{array}$$ (15)

### 3.3 Asymptotic confidence interval (ACI) based on MLE

In this section, we use the delta approach to calculate the ACI of SSR *ϕ* based ML estimators since the exact distribution of $\hat{\phi }$ is unavailable. Let $\hat{\psi }=\left({\hat{\alpha }}_{1},{\hat{\alpha }}_{2}\right)$ be the ML estimates of *ψ* = (*α*_1_, *α*_2_). The asymptotic variance of $\hat{\psi }$ using delta method, see [24], is given by

$$\begin{array}{c} Var\left(\hat{\psi }\right)=\left[q^{\prime }I^{-1}\left(\psi \right)q\right], \end{array}$$

where, $I\left(\psi \right)=-E\left[\begin{array}{cc} \frac{\partial^{2}l\left(\alpha_{1},\alpha_{2}\right)}{\partial \alpha_{1}^{2}} & \frac{\partial^{2}l\left(\alpha_{1},\alpha_{2}\right)}{\partial \alpha_{1}\partial \alpha_{2}}\\ \frac{\partial^{2}l\left(\alpha_{1},\alpha_{2}\right)}{\partial \alpha_{2}\partial \alpha_{1}} & \frac{\partial^{2}l\left(\alpha_{1},\alpha_{2}\right)}{\partial \alpha_{2}^{2}} \end{array}\right]$ is the Fisher information matrix (FIM) and $q={\left(\frac{\partial \phi }{\partial \alpha_{1}},\frac{\partial \phi }{\partial \alpha_{2}}\right)}^{\prime }.$

The observed FIM can be utilized as a consistent estimator of the Fisher information under modest regularity criteria. Thus, the observed variance of $\hat{\phi }$ is given by

$$\hat{V}ar\left(\hat{\phi }\right)≃{\left[q^{\prime }I^{-1}\left(\psi \right)q\right]}_{\psi =\hat{\psi }}.$$

In the FIM *I*(*ψ*), the partial derivative elements are provided by

$$\begin{array}{c} \frac{\partial^{2}l\left(\alpha_{1},\alpha_{2}\right)}{\partial \alpha_{1}^{2}}=-\frac{m_{1}}{\alpha_{1}^{2}}-\sum_{i=1}^{m_{1}}\;\left[k_{1}\left(G_{i}+1\right)-1\right]\frac{{\left[\;\text{ln}\;\left(\frac{x_{i}}{1+x_{i}}\right)\right]}^{2}{\left(\frac{x_{i}}{1+x_{i}}\right)}^{\alpha_{1}}}{{\left[1-{\left(\frac{x_{i}}{1+x_{i}}\right)}^{\alpha_{1}}\right]}^{2}}, \end{array}$$

$$\begin{array}{c} \frac{\partial^{2}l\left(\alpha_{1},\alpha_{2}\right)}{\partial \alpha_{2}^{2}}=-\frac{m_{2}}{\alpha_{2}^{2}}-\sum_{j=1}^{m_{2}}\;\left[k_{2}\left(W_{j}+1\right)-1\right]\frac{{\left[\;\text{ln}\;\left(\frac{y_{j}}{1+y_{j}}\right)\right]}^{2}{\left(\frac{y_{j}}{1+y_{j}}\right)}^{\alpha_{2}}}{{\left[1-{\left(\frac{y_{j}}{1+y_{j}}\right)}^{\alpha_{1}}\right]}^{2}}, \end{array}$$

$$\begin{array}{c} \frac{\partial^{2}l\left(\alpha_{1},\alpha_{2}\right)}{\partial \alpha_{1}\partial \alpha_{2}}=\frac{\partial^{2}l\left(\alpha_{1},\alpha_{2}\right)}{\partial \alpha_{2}\partial \alpha_{1}}=0, \end{array}$$

and the elements of *q* are given by

$$\begin{array}{c} \frac{\partial \phi }{\partial \alpha_{1}}=\frac{\alpha_{2}}{{\left(\alpha_{1}+\alpha_{2}\right)}^{2}},\;\;\;\frac{\partial \phi }{\partial \alpha_{2}}=-\frac{\alpha_{1}}{{\left(\alpha_{1}+\alpha_{2}\right)}^{2}}. \end{array}$$

Thus $\frac{\hat{\phi }-\phi }{\sqrt{\hat{V}ar\left(\hat{\phi }\right)}}∼N\left(0,1\right)$. Therefore, the 100(1 − *ξ*)% ACI of *ϕ* is given by $\hat{\phi }\pm z_{\xi /2}\sqrt{\hat{V}ar\left(\hat{\phi }\right)}$, where *z*_*ξ*/2_ is the upper (*ξ*/2)^*th*^ quantile of *N*(0, 1).

## 4 Bayesian estimation

Here, we compute the Bayes estimator of SSR *ϕ* under SELF. Assume that the unknown parameter *α*_1_ and *α*_1_ have gamma distribution with the following pdfs, respectively

$$\begin{array}{c} h_{1}\left(\alpha_{1}\right)\propto \alpha_{1}^{r_{1}-1}\;\text{exp}\;\left(-s_{1}\alpha_{1}\right);\;\;\alpha_{1}>0,r_{1},s_{1}>0, \end{array}$$

and

$$\begin{array}{c} h_{2}\left(\alpha_{2}\right)\propto \alpha_{2}^{r_{2}-1}\;\text{exp}\;\left(-s_{2}\alpha_{2}\right);\;\;\alpha_{2}>0,r_{2},s_{2}>0, \end{array}$$

where *r*_*i*_, *s*_*i*_; *i* = 1, 2 are hyper-parameters selected to represent previous knowledge of the parameters *α*_1_ and *α*_2_, respectively. Therefore, the joint prior distribution of *α*_1_ and *α*_2_ can be defined as

$$\begin{array}{c} h\left(\alpha_{1},\alpha_{2}\right)\propto \alpha_{1}^{r_{1}-1}\alpha_{2}^{r_{2}-1}\;\text{exp}\;\left\{-\left(s_{1}\alpha_{1}+s_{2}\alpha_{2}\right)\right\}. \end{array}$$ (16)

The choice of gamma priors is not unreasonable, as the family of gamma distributions is quite diverse, with many different types of distributions. Independent gamma priors are specific examples of non-informative priors. Many researchers have used gamma priors in a variety of situations, such as [25, 26], etc. Now, by incorporating the joint prior (16) to the likelihood function (11), the posterior distribution of *α*_1_ and *α*_2_ is given by

$$\begin{array}{cc} \pi \left(\alpha_{1},\alpha_{2}|\underset{˜}{x},\underset{˜}{y}\right) & =\frac{L\left(\alpha_{1},\alpha_{2};\underset{˜}{x},\underset{˜}{y}\right)h\left(\alpha_{1},\alpha_{2}\right)}{\int_{0}^{\infty }\int_{0}^{\infty }L\left(\alpha_{1},\alpha_{2};\underset{˜}{x},\underset{˜}{y}\right)h\left(\alpha_{1},\alpha_{2}\right)d\alpha_{1}d\alpha_{2}}\\ \Rightarrow \pi \left(\alpha_{1},\alpha_{2}|\underset{˜}{x},\underset{˜}{y}\right) & \propto \alpha_{1}^{m_{1}+r_{1}-1}\alpha_{2}^{m_{2}+r_{2}-1}\;\text{exp}\;\{-\alpha_{1}\left[s_{1}-\sum_{i=1}^{m_{1}}\;\text{ln}\;\left(\frac{x_{i}}{1+x_{i}}\right)\right]\}\\ & \times \;\text{exp}\;\{-\alpha_{2}\left[s_{2}-\sum_{j=1}^{m_{2}}\;\text{ln}\;\left(\frac{y_{j}}{1+y_{j}}\right)\right]\}\\ & \prod_{i=1}^{m_{1}}{\left[1-{\left(\frac{x_{i}}{1+x_{i}}\right)}^{\alpha_{1}}\right]}^{k_{1}\left(G_{i}+1\right)-1}\prod_{j=1}^{m_{2}}{\left[1-{\left(\frac{y_{j}}{1+y_{j}}\right)}^{\alpha_{2}}\right]}^{k_{2}\left(W_{j}+1\right)-1}. \end{array}$$ (17)

We use one of the Markov Chain Monte Carlo (MCMC) techniques, the Metropolis-Hastings (M-H) algorithm, to compute the Bayes estimate and the accompanying HPD credible interval of SSR *ϕ*, as the posterior distribution in Eq (17) cannot be determined analytically.

### 4.1 Metropolis-Hastings algorithm

Here, the Bayes estimator and HPD credible interval of SSR *ϕ* are created using the M-H algorithm. The M-H method is a widely used MCMC approach for obtaining random samples from any arbitrarily complicated target distribution of any dimension that is known up to a normalizing constant. [27] for further information on MCMC approaches and their applications. The marginal posterior distributions of *α*_1_ and *α*_2_ can be defined as follows:

$$\begin{array}{cc} \pi_{1}\left(\alpha_{1}|\alpha_{2},\underset{˜}{x},\underset{˜}{y}\right) & \propto \alpha_{1}^{m_{1}+r_{1}-1}\;\text{exp}\;\{-\alpha_{1}\left[s_{1}-\sum_{i=1}^{m_{1}}\;\text{ln}\;\left(\frac{x_{i}}{1+x_{i}}\right)\right]\}\\ & \;\;\;\;\prod_{i=1}^{m_{1}}{\left[1-{\left(\frac{x_{i}}{1+x_{i}}\right)}^{\alpha_{1}}\right]}^{k_{1}\left(G_{i}+1\right)-1} \end{array}$$ (18)

and

$$\begin{array}{cc} \pi_{2}\left(\alpha_{2}|\alpha_{1},\underset{˜}{x},\underset{˜}{y}\right) & \propto \alpha_{2}^{m_{2}+r_{2}-1}\;\text{exp}\;\{-\alpha_{2}\left[s_{2}-\sum_{j=1}^{m_{2}}\;\text{ln}\;\left(\frac{y_{j}}{1+y_{j}}\right)\right]\}\\ & \;\;\;\;\prod_{j=1}^{m_{2}}{\left[1-{\left(\frac{y_{j}}{1+y_{j}}\right)}^{\alpha_{2}}\right]}^{k_{2}\left(W_{j}+1\right)-1}. \end{array}$$ (19)

Since the marginal posterior distributions of *α*_1_ and *α*_2_ are not well-known, the M–H algorithm can be used to generate random numbers from these distributions. In this situation, the proposal density is based on the normal distribution. Consequently, to sample from the marginal posteriors, the following steps are used:

Step 1: Begin with an initial guess ($\alpha_{1}^{\left(0\right)}$, $\alpha_{2}^{\left(0\right)}$).
Step 2: Set *t* = 1.
Step 3: Using the M-H algorithm with normal proposal density, generate $\alpha_{1}^{\left(t\right)}$ from $\pi_{1}\left(\alpha_{1}|\alpha_{2},\underset{˜}{x},\underset{˜}{y}\right)$ in Eq (18).
Step 4: Using the M-H algorithm with normal proposal density, generate $\alpha_{2}^{\left(t\right)}$ from $\pi_{2}\left(\alpha_{2}|\alpha_{1},\underset{˜}{x},\underset{˜}{y}\right)$ in Eq (19).
Step 5: Compute $\phi^{\left(t\right)}=\phi \left(\alpha_{1}^{\left(t\right)},\alpha_{2}^{\left(t\right)}\right)$ using Eq (4).
Step 6: Set *t* = *t* + 1.
Step 7: Repeat steps 3 to 6, (*M* − 1) times.

Now, the Bayes estimate ${\hat{\phi }}_{Bayes}$ of SSR under SELF is the posterior mean and it is obtained as

$$\begin{array}{c} {\hat{\phi }}_{\text{Bayes}}=\hat{E}\left(\phi |\text{data}\right)=\frac{1}{M-M_{0}}\sum_{t=M_{0}+1}^{M}\phi^{\left(t\right)}. \end{array}$$ (20)

We discarded the observations $\phi^{\left(1\right)},\phi^{\left(2\right)},\ldots ,\phi^{\left(M_{0}\right)}$, worked with (*M* − *M*_0_) remaining observations, which are seen as an independent sample from the stationary distribution of the Markov chain, which is generally the posterior distribution.

### 4.2 HPD credible interval

Here, using the generated MCMC samples, we obtained the HPD credible interval of SSR *ϕ* with the help of [28] algorithm. Let *ϕ*_(1)_ < *ϕ*_(2)_ < ⋯ < *ϕ*_(*M*)_ be the ordered values of *ϕ*^(1)^, *ϕ*^(2)^, …, *ϕ*^(*M*)^. Then, 100(1 − *ξ*)%, HPD credible interval of SSR *ϕ* is given by (*ϕ*_(*t*)_, *ϕ*_(*t*+[(1−*ξ*)*M*])_), where *t* is chosen such that

$$\phi_{\left(t+\left[\left(1-\xi \right)M\right]\right)}-\phi_{\left(t\right)}=\underset{1\leq i\leq \xi M}{\text{min}}\;\left(\phi_{\left(i+\left[\left(1-\xi \right)M\right]\right)}-\phi_{\left(t\right)}\right),t=1,2,\ldots ,M,$$

where, [*x*] is the integral part of *x*.

## 5 Monte Carlo simulation

The efficiency of the various estimators covered in this work is examined using a comprehensive Monte Carlo simulation study. The average values (AV) and mean squared errors (MSE) are used to compare these estimators (MSE). In addition, the interval estimates are compared with average lengths (AL). The Bayes estimate of SSR *ϕ* is obtained under SELF by incorporating gamma prior distributions. The following steps are carried out for the simulations as follows:

1. Considered number of groups *n* = *n*_1_ = *n*_2_ with same group sizes *k* = *k*_1_ = *k*_2_. Also, we assumed same prefixed number of failures *m* = *m*_1_ = *m*_2_ with same prefixed censoring schemes $CS=\underset{˜}{G}=\underset{˜}{W}$.
2. The different sample sizes with different prefixed censoring plans for computation purposes are tabulated in Table 1. The simplified notations used in Table 1 like for CS = 1, (5 * 1, 0 * 19) denotes (5, 0, 0, 0, 0, 0, 0, 0, 0, 0, 0, 0, 0, 0, 0, 0, 0, 0, 0, 0).
3. Two sets of true parameters (*α*_1_, *α*_2_) = (2, 0.5) and (*α*_1_, *α*_2_) = (1.2, 0.8) are chosen. Consequently, the corresponding true values of SSR *ϕ* become, *ϕ* = 0.80 and *ϕ* = 0.60, respectively.
4. Generate $\underset{˜}{x}$ and $\underset{˜}{y}$ independent PFFC samples with effective sample sizes *m*, using [2] algorithm with distribution function 1 − (1 − *F*(.))^*k*^ from IPD.
5. Compute MPS and ML estimates of SSR *ϕ*. Also, compute the ACI of SSR based on ML estimators.
6. For Bayesian computations, the hyper-parameters are chosen as {(*r*_1_, *s*_1_) = (4, 2), (*r*_2_, *s*_2_) = (2, 4)} and {(*r*_1_, *s*_1_) = (2.4, 2), (*r*_2_, *s*_2_) = (3.2, 4)} with the assumption that the prior means equal to true parameter values, respectively. *M* = 10, 000 posterior samples are generated using M-H algorithm, and *M*_0_ = 2, 000 samples are discarded as burn-in-period. Then, compute Bayes estimate and HPD credible interval of SSR *ϕ*.
7. Run the whole process 1000 times and take the average values of the estimates.

**Table 1:** Different censoring schemes used in the simulation study.

*n*	*m*	CS	Scheme	*n*	*m*	CS	Scheme
25	20	1	(5 * 1,0 * 19)	35	30	6	(0 * 29, 5)
25	20	2	(1 * 2, 0 * 8, 1 * 1, 0 * 7, 1 * 2)	50	40	7	(10 * 1, 0 * 39)
25	20	3	(0 * 19,5)	50	40	8	(1 * 5, 0 * 30, 1 * 5)
35	30	4	(5 * 1, 0 * 29)	50	40	9	(0 * 39, 10 * 1)
35	30	5	(1 * 2, 0 * 12, 1 * 1, 0 * 13, 1 * 2)				

In Tables 2 and 3, all of the simulated outcomes are shown. Following are the conclusions drawn from these simulation tables:

**Table 2:** The MPS, ML and Bayes estimates of SSR *ϕ*, when *ϕ* = 0.80.

		${\tilde{\phi }}_{\text{MPS}}$		${\hat{\phi }}_{\text{ML}}$			${\hat{\phi }}_{\text{MH}}$		
(*n*, *m*, *k*)	CS	AV	MSE	AV	MSE	AL	AV	MSE	AL
(25,20,2)	1	0.7961	0.0015	0.7960	0.0014	0.1433	0.7958	0.0015	0.0305
(25,20,2)	2	0.7961	0.0014	0.7961	0.0014	0.1367	0.7950	0.0013	0.0927
(25,20,2)	3	0.7990	0.0011	0.7990	0.0011	0.1319	0.7981	0.0011	0.1162
(35,30,2)	4	0.7977	0.0010	0.7976	0.0010	0.1184	0.7971	0.0010	0.0179
(35,30,2)	5	0.7987	0.0009	0.7987	0.0009	0.1140	0.7980	0.0008	0.0752
(35,30,2)	6	0.7984	0.0010	0.7984	0.0009	0.1117	0.7978	0.0009	0.0961
(50,40,2)	7	0.7975	0.0007	0.7975	0.0007	0.1022	0.7974	0.0007	0.0128
(50,40,2)	8	0.7984	0.0007	0.7984	0.0007	0.0966	0.7978	0.0007	0.0652
(50,40,2)	9	0.7992	0.0006	0.7992	0.0006	0.0935	0.7988	0.0006	0.0828
(25,20,4)	1	0.7970	0.0013	0.7977	0.0011	0.1430	0.7972	0.0011	0.0304
(25,20,4)	2	0.7988	0.0009	0.7981	0.0009	0.1347	0.7981	0.0008	0.0916
(25,20,4)	3	0.7981	0.0008	0.7976	0.0008	0.1315	0.7977	0.0007	0.1164
(35,30,4)	4	0.7993	0.0005	0.7987	0.0005	0.1169	0.7993	0.0005	0.0174
(35,30,4)	5	0.7991	0.0004	0.7985	0.0004	0.1130	0.7987	0.0004	0.0750
(35,30,4)	6	0.8000	0.0004	0.7994	0.0004	0.1103	0.7997	0.0004	0.0953
(50,40,4)	7	0.7988	0.0003	0.7983	0.0003	0.1009	0.7994	0.0003	0.0121
(50,40,4)	8	0.7990	0.0002	0.7984	0.0002	0.0956	0.7988	0.0002	0.0650
(50,40,4)	9	0.7990	0.0001	0.7984	0.0001	0.0928	0.7991	0.0001	0.0828

**Table 3:** The MPS, ML and Bayes estimates of SSR *ϕ*, when *ϕ* = 0.60.

	${\tilde{\phi }}_{\text{MPS}}$		${\hat{\phi }}_{\text{ML}}$			${\hat{\phi }}_{\text{MH}}$		
CS	AV	MSE	AV	MSE	AL	AV	MSE	AL
1	0.5957	0.0033	0.5957	0.0033	0.2114	0.5951	0.0033	0.0393
2	0.6012	0.0029	0.6012	0.0029	0.2012	0.6010	0.0027	0.1352
3	0.5998	0.0027	0.5998	0.0026	0.1964	0.5992	0.0024	0.1719
4	0.5997	0.0022	0.5997	0.0022	0.1756	0.5995	0.0023	0.0273
5	0.5985	0.0020	0.5985	0.0020	0.1698	0.5985	0.0019	0.1109
6	0.5995	0.0020	0.5996	0.0020	0.1661	0.5991	0.0019	0.1421
7	0.5974	0.0016	0.5974	0.0016	0.1519	0.5976	0.0017	0.0212
8	0.5980	0.0014	0.5980	0.0014	0.1440	0.5981	0.0014	0.0968
9	0.5981	0.0015	0.5970	0.0015	0.1442	0.5982	0.0013	0.0969
1	0.6017	0.0019	0.6017	0.0019	0.1626	0.6015	0.0020	0.0290
2	0.6015	0.0017	0.6015	0.0017	0.1551	0.6014	0.0016	0.1058
3	0.6020	0.0016	0.6020	0.0016	0.1509	0.6015	0.0015	0.1316
4	0.5995	0.0012	0.5995	0.0012	0.135	0.5990	0.0012	0.0222
5	0.5980	0.0012	0.5980	0.0012	0.1305	0.5979	0.0012	0.0865
6	0.6004	0.0012	0.6004	0.0012	0.1274	0.6001	0.0011	0.1088
7	0.6013	0.0009	0.6013	0.0009	0.1164	0.6015	0.0010	0.0181
8	0.6007	0.0009	0.6007	0.0009	0.1105	0.6007	0.0009	0.0748
9	0.6004	0.0008	0.6004	0.0008	0.1071	0.6002	0.0008	0.0939

The outcomes of MPS, ML, and Bayes estimates of SSR in terms of AV and MSEs are very adequate, even for small sample sizes in almost all cases. As *n* and *m* increases, the MSEs decline, confirming the consistency of different SSR estimators. Additionally, the MSEs drop as the number of test units in a group grows. In terms of MSEs, the Bayes estimator outperforms the ML and MPS estimators because Bayes estimators take into account previous information about the parameters. In addition, the performance of the MPS estimator is quite better than that of the ML estimator in terms of MSEs. In addition, when the number of failures increases, the ALs of ACI and HPD credible intervals decline. It is also observed that HPD credible intervals exhibit smaller ALs than ACIs. As a result, we may infer that the Bayes estimator works substantially better when prior information is available, and that it can be utilized for any practical purpose. Also, we find that the censoring scheme 9 provides the best results for classical as well as for Bayesian estimation methods.

## 6 Real life applications

This section discusses real-life applications for the illustrations of the proposed methodology developed in this study. For this purpose, two pairs of real data sets are considered and analyzed in the following subsections:

### 6.1 Jute fibres’ breaking strength data

[29] discussed the breaking strength of jute fibres at four different gauge lengths as 5 mm, 10 mm, 15 mm, and 20 mm, respectively. Here, we consider the breaking strength of jute fibres at two distinct gauge lengths 15 mm (say *X*_1_) and 20 mm (say *Y*_1_) by dividing each observation by 10. The transformed data sets are given by

*X*_1_ (15 mm): 59.440, 20.275, 16.837, 57.486, 22.565, 7.638, 15.667, 12.781, 81.387, 56.239, 46.847, 13.509, 7.224, 49.794, 35.556, 56.907, 64.048, 20.042, 55.042, 74.875, 48.966, 67.806, 45.771, 10.673, 71.630, 4.266, 8.040, 33.922, 7.009, 19.342.
*Y*_1_ (20 mm): 7.146, 41.902, 28.464, 58.557, 45.660, 11.385, 18.785, 68.816, 66.266, 4.558, 57.862, 75.670, 59.429, 16.649, 9.972, 70.736, 76.514, 18.713, 14.596, 35.070, 54.744, 11.699, 37.581, 58.160, 11.986, 4.801, 20.016, 3.675, 24.453, 8.355.

To begin, we examine the goodness of fit to see if the IPD can be used to analyze these data sets separately. The Kolmogorov-Smirnov (KS) statistics along with associated *p*-values based on ML estimates are computed. The ML estimates of unknown parameters *α*_1_ and *α*_2_ are computed as 19.2748 and 16.4732, respectively. The KS statistics (*p*-values) are computed as 0.2097 (0.1232) and 1926 (0.1894), respectively. Based on the *p*-values, we can say that the considered data sets are good fits for the IPD model. Further, after randomly grouping the considered data sets into *n* = 15 groups with *k* = 2 items within each group, and then consider the first failure censored samples. The bold observation shows the first-failure observation in the respective groups as shown in Table 4.

**Table 4:** First-failure censored jute fibres’ breaking strength data.

Data Sets	Groups Items	1	2	3	4	5	6	7	8
*X*_1_(15*mm*)	i ii	75.67	**35.07**	58.557	**4.801**	**11.699**	28.464	20.016	**14.596**
		**16.649**	54.744	**8.355**	7.146	66.266	**4.558**	**18.713**	18.785
		9	10	11	12	13	14	15	
		45.66	76.514	**3.675**	**24.453**	**11.385**	57.862	**37.581**	
		**9.972**	**70.736**	68.816	41.902	58.16	**11.986**	59.429	
	Groups Items	1	2	3	4	5	6	7	8
*Y*_1_(20*mm*)	i ii	58.16	**11.699**	**9.972**	68.816	14.596	**4.801**	**4.558**	58.557
		**41.902**	75.67	70.736	**45.66**	**7.146**	11.986	11.385	**54.744**
		9	10	11	12	13	14	15	
		**16.649**	**35.07**	66.266	**3.675**	**18.785**	20.016	59.429	
		37.851	76.514	**28.464**	57.862	24.453	**18.713**	**8.355**	

Finally, the first failure-censored samples of the considered data sets, respectively, are given by

*X*_1_ (15 mm): 4.266, 7.009, 7.224, 8.040, 10.673, 13.509, 15.667, 16.837, 19.342, 20.042, 20.275, 45.771, 46.847, 49.794, 56.239.
*Y*_1_ (20 mm): 3.675, 4.558, 4.801, 8.355, 9.972, 11.699, 14.596, 18.713, 18.785, 20.016, 24.453, 28.464, 37.581, 54.744, 59.429.

Now, applying four different common prefixed censoring schemes on the above ordered first failure censored samples with effective sample size *m* = 10. The four different common prefixed censoring schemes and their associated PFFC samples are as follows:

Scheme 1: $k=2,\;n=15,\;m=10,\;\underset{˜}{W}=\left(5,0,0,0,0,0,0,0,0,0\right)$,

${\underset{˜}{x}}_{1}=4.266$ , 12.781, 13.509, 16.837, 19.342, 20.042, 22.565, 46.847, 57.486, 71.630.

${\underset{˜}{y}}_{1}=3.675$ , 11.385, 11.699, 11.986, 16.649, 24.453, 28.464, 45.660, 58.160, 59.429.
Scheme 2: $k=2,\;n=15,\;m=10,\;\underset{˜}{W}=\left(1,0,0,1,0,0,2,0,0,1\right)$,

${\underset{˜}{x}}_{1}=4.266$ , 7.224, 7.638, 8.040, 12.781, 13.509, 16.837, 22.565, 46.847, 48.966.

${\underset{˜}{y}}_{1}=3.675$ , 4.801, 7.146, 8.355, 11.385, 11.986, 14.596, 24.453, 28.464, 37.581.
Scheme 3: $k=2,\;n=15,\;m=10,\;\underset{˜}{W}=\left(0,0,0,0,0,0,0,0,0,5\right)$,

${\underset{˜}{x}}_{1}=4.266$ , 7.009, 7.224, 7.638, 8.040, 12.781, 13.509, 16.837, 19.342, 20.275.

${\underset{˜}{y}}_{1}=3.675$ , 4.558, 4.801, 7.146, 9.972, 11.385, 11.699, 11.986, 16.649, 20.016.
Scheme 4: $k=2,\;n=m=15,\;\underset{˜}{W}=\left(0,0,0,0,0,0,0,0,0,0,0,0,0,0,0\right)$,

${\underset{˜}{x}}_{1}=4.266$ , 7.009, 7.224, 7.638, 8.040, 10.673, 12.781, 13.509, 15.667, 19.342, 20.042, 22.565, 46.847, 49.794, 64.048.

${\underset{˜}{y}}_{1}=3.675$ , 4.558, 4.801, 8.355, 9.972, 11.385, 11.986, 16.649, 18.713, 18.785, 24.453, 35.070, 41.902, 57.862, 66.266.

**Fig 1:**
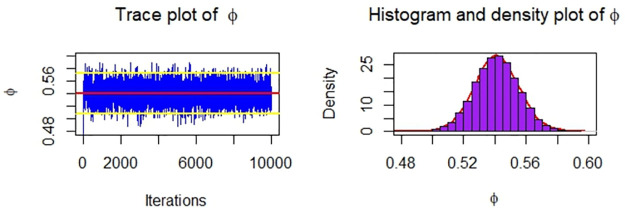
MCMC trace, and plot of the posterior distribution of *ϕ* for scheme 1 under consideration of jute fibres’ breaking strength data.

**Fig 2:**
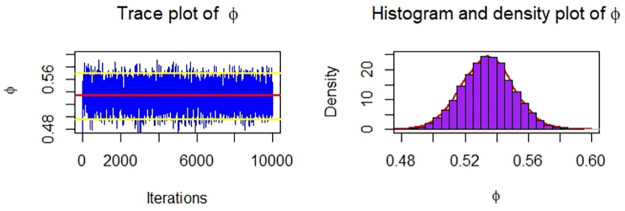
MCMC trace, and plot of the posterior distribution of *ϕ* for Scheme 2 under consideration of jute fibres’ breaking strength data.

**Fig 3:**
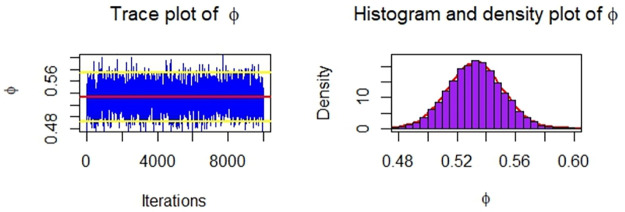
MCMC trace, and plot of the posterior distribution of *ϕ* for Scheme 3 under consideration of jute fibres’ breaking strength data.

**Fig 4:**
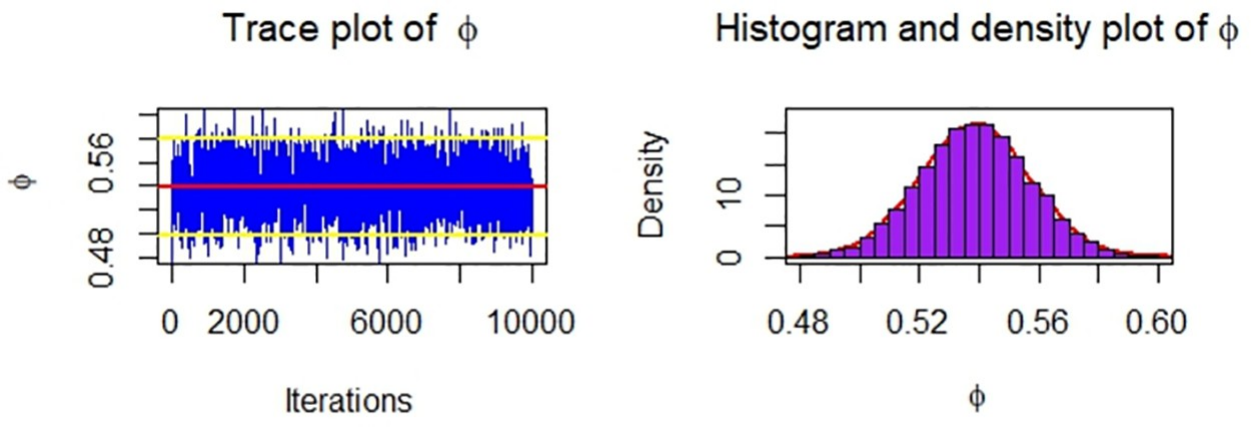
MCMC trace, and plot of the posterior distribution of *ϕ* for Scheme 4 under consideration of jute fibres’ breaking strength data.

Now, we obtain the ML, MPS, and Bayes estimates of SSR under consideration of four different censoring schemes. Also, computed asymptotic confidence and HPD credible intervals of SSR, see, Table 5. For the Bayesian computation, the hyper-parameters are taken as *r*_*i*_ = *s*_*i*_ = 0.0001; *i* = 1, 2 as we don’t have any prior information. We generate 10,000 posterior samples from the marginal posteriors (18) and (19) using the M-H algorithm. Trace plots and posterior distribution plots for the jute fibres’ breaking strength data are given in the following Figs 1–4, respectively. These plots demonstrate the feasibility of MCMC techniques.

**Table 5:** The MPS, ML, and Bayes estimates of the SSR (asymptotic and HPD CIs) for the jute fibres’ breaking strength data.

Schemes	MPS	MLE	ACI	Bayes	HPD
Schemes 1	0.5670	0.5403	(0.3687, 0.7119)	0.5775	(0.4914, 0.6083)
Schemes 2	0.5529	0.5521	(0.3660, 0.6505)	0.5518	(0.4484, 0.6598)
Schemes 3	0.5372	0.5445	(0.4140, 0.7224)	0.5442	(0.4379, 0.6511)
Schemes tab: analysis 0.5533	(0.4050, 0.7017)	0.5087	(0.3981, 0.6115)		

### 6.2 Electrical insulation data

In this illustration, two different types of electrical insulation failure times (measured in seconds) under continuous-increasing voltage stress are considered. Two electrical insulation’s are tested and recorded each of size 30. These data are studied by [1]. Here, we consider these data after multiplying each observation by 10. The transformed failure times of two different electrical insulation’s each of 30 sizes, respectively, are as follows:

*X*_2_ (in seconds): 0.97, 0.14, 0.3, 1.34, 2.4, 0.84, 1.46, 0.24, 0.45, 0.04, 0.99, 2.77, 4.72, 0.94, 0.23, 1.46, 0.3, 0.31, 1.04, 1.05, 0.36, 0.65, 0.22, 0.98, 1.78, 0.59, 0.14, 0.07, 0.07, 2.86.

*Y*_2_ (in seconds): 1.99, 2.52, 1.03, 4.55, 1.35, 3.48, 3.21, 1.66, 0.4, 0.27, 5.19, 2.7, 0.08, 0.3, 0.84, 2.36, 3.15, 1.77, 2.68, 1.8, 7.96, 2.45, 7.03, 0.45, 0.17, 8.21, 9.42, 3.14, 2.81, 6.52.

**Fig 5:**
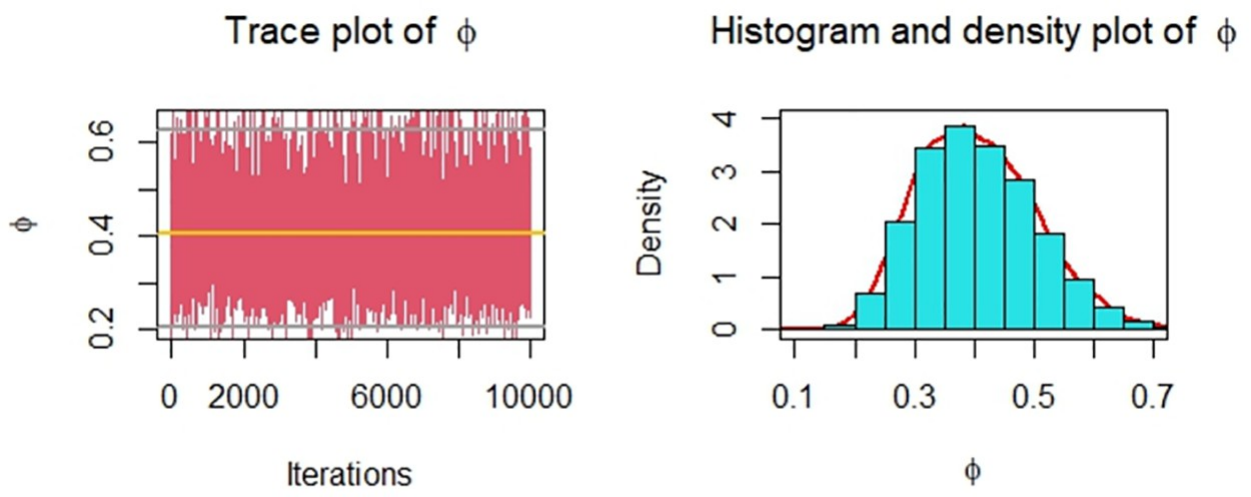
MCMC trace, and plot of the posterior distribution of *ϕ* for Scheme 1 under consideration of electrical insulation data.

**Fig 6:**
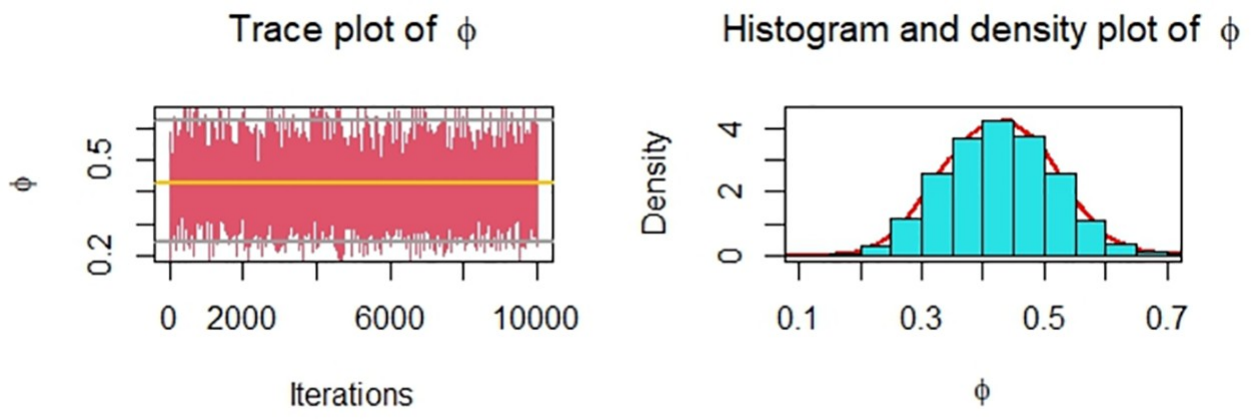
MCMC trace, and plot of the posterior distribution of *ϕ* for Scheme 2 under consideration of electrical insulation data.

**Fig 7:**
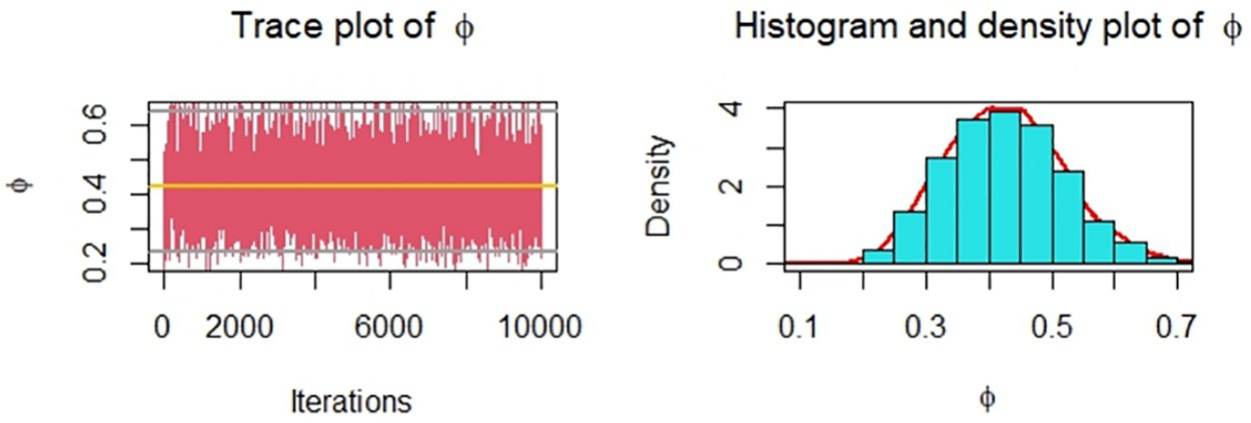
MCMC trace, and plot of the posterior distribution of *ϕ* for Scheme 3 under consideration of electrical insulation data.

**Fig 8:**
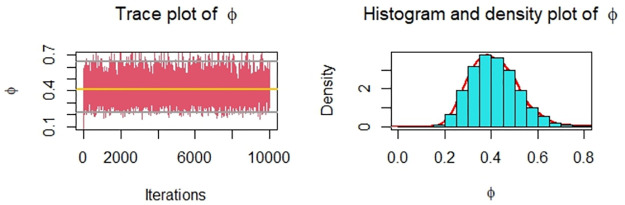
MCMC trace, and plot of the posterior distribution of *ϕ* for Scheme 4 under consideration of electrical insulation data.

First, we check whether IPD fits these data sets. We find KS statistics along with associated *p*-value based on ML estimates are computed. The ML estimates of ${\hat{\alpha }}_{1}$ and ${\hat{\alpha }}_{2}$ are 0.8598 and 1.6871 respectively. KS distance along with p-values are 0.1948 (0.2050) and 0.1565(0.4120) respectively. According to the p-values, we can say that IPD fits well for these data sets. As discussed in the sub-section (6.1), we make four progressively first failure censored samples with effective sample size *m* = 8, which are tabulated in Table 6 along with four different progressively first failure censoring schemes (CS). Now obtain the ML, MPS, Bayes estimates, asymptotic confidence, and HPD credible intervals of SSR under consideration of four different censoring schemes. The obtained results are reported in Table 7. The trace plots and posterior density plots for the electrical insulation data are given in Figs 5–8. These plots show that MCMC methods perform well.

**Table 6:** Different censoring schemes (CS) and their corresponding PFFC samples for the electrical insulation data.

(*k*, *n*, *m*)	CS	Schemes	PFFC samples
(3,10,8)	1	(2, 0,0,0,0,0,0,0)	${\underset{˜}{x}}_{2}=0.04$ , 0.22, 0.23, 0.30, 0.30, 0.36, 0.59, 0.65
			${\underset{˜}{y}}_{2}=0.08$ , 0.30, 0.40, 0.45, 0.84, 1.03, 2.68, 3.15.
(3,10,8)	2	(1,0,0,0,0,0,0,1)	${\underset{˜}{x}}_{2}$ = 0.04, 0.14, 0.22, 0.23, 0.24, 0.31, 0.45, 0.97.
			${\underset{˜}{y}}_{2}=0.08$ , 0.27, 0.40, 0.84, 1.03, 1.35, 1.66, 2.68.
(3,10,8)	3	(0,0,0,0,0,0,0,2)	${\underset{˜}{x}}_{2}=0.04$ , 0.07, 0.07, 0.14, 0.14, 0.23, 0.31, 0.45.
			${\underset{˜}{y}}_{2}=0.08$ , 0.17, 0.27, 0.30, 0.84, 1.03, 1.66, 1.80.
(3,10,10)	4	(0,0,0,0,0,0,0,0,0,0)	${\underset{˜}{x}}_{2}=0.04$ , 0.07, 0.07, 0.14, 0.14, 0.22, 0.23, 0.24, 0.30, 0.45.
			${\underset{˜}{y}}_{2}=0.08$ , 0.17, 0.27, 0.30, 0.40, 0.45, 1.35, 2.36, 2.52, 2.68

**Table 7:** MPS, ML, and Bayes estimates of SSR *ϕ* (asymptotic and HPD CIs) for the electrical insulation data.

Schemes	MPS	MLE	ACI	Bayes	HPD
Schemes 1	0.3461	0.3520	(0.2414, 0.4626)	0.3617	(0.1701, 0.6142)
Schemes 2	0.4112	0.4012	(0.2835, 0.5189)	0.4253	(0.2439, 0.6256)
Schemes 3	0.4221	0.4111	(0.2946, 0.5276)	0.4238	(0.2377, 0.6411)
Schemes 4	0.3991	0.3989	(0.2861, 0.5117)	0.4102	(0.2138, 0.6432)

## 7 Conclusion

The concept of estimating SSR for IPD using PFFC samples from both the classical and Bayesian prospective was tackled in this study. In the case of the classical estimation procedure, two estimation methods, the ML and MPS methods, are used to estimate SSR. The MPS method for PFFC data has not yet been discussed in the literature. Also, 95% of asymptotic confidence and HPD credible intervals of SSR were constructed. Extensive simulations were examined to see the performance of different estimation procedures. The outcomes of the simulation results suggest that the Bayes estimator is more precise than the ML and MPS estimators. In addition, the performance of the MPS estimator is quite better than the ML estimator. Thus, for all practical purposes, the Bayes estimator can be a good choice when the prior information is available; otherwise, the MPS or the ML method is commended. Finally, to demonstrate the methodologies considered in this study, we analyzed two different pairs of real data sets as illustrative examples. The approach and estimation results presented in this paper will be valuable to reliability practitioners in real-world situations.
